# Pasteurism

**Published:** 1898-08

**Authors:** Montague R. Leverson

**Affiliations:** Fort Hamilton, N. Y.


### PASTEURISM.

FORT HAMILTON, N. Y., June 24th, 1898.

TO THE EDITOR OF THE HOMŒOPATHIC PHYSICIAN.

DEAR SIR:—In the journal *Le Medicine*, of Brussels, of the 12th inst., quoting the *Journal de Medicine*, of Paris, is given an account of a policeman of that city having been badly bitten by a mad dog. The next day he went to the Pasteur Institute, where for eighteen days he was treated, and rigorously followed the prescribed treatment, *and was discharged cured!* "Last week," says the Paris medical journal, "he felt certain pains, which excited the suspicions of the doctor whom he consulted. That gentleman telephoned to the Director of the Pasteur Institute, who replied that his trouble must be attributed to some other cause than hydrophobia, *because he had left their institute completely cured.* At the end of two or three days doubt was no longer possible, the case was clearly one of hydrophobia. He was taken to the hospital, where he died last Sunday morning in frightful convulsions."

This case will doubtless figure in the annals of the Pasteur institutes as a cure!

I have the honor to be, sir,

Yours respectfully,

MONTAGUE R. LEVERSON.

---

MR. TEBB'S CONTRIBUTIONS TO THE VACCINATION TESTIMONY.—Owing to the requests from English readers it has been decided to somewhat change the order of publication of the Vaccination testimony, and instead of giving Dr. Creighton's testimony which is now next in order, in its place to give that of Mr. Tebb which is equally interesting.—[Ed.]

